# Mindfulness and mood stimulate each other in an upward spiral: a mindful walking intervention using experience sampling

**DOI:** 10.1007/s12671-016-0550-8

**Published:** 2016-06-02

**Authors:** Rinske A. Gotink, Karlijn S.F.M. Hermans, Nicole Geschwind, Reinier De Nooij, Wouter T. De Groot, Anne E.M. Speckens

**Affiliations:** 1 Department of Epidemiology, Erasmus MC Rotterdam, Rotterdam, Netherlands; 2 Department of Clinical Psychology Science, Maastricht University, Maastricht, Netherlands; 3 Institute for Science, Innovation and Society, Radboud University Nijmegen, Nijmegen, Netherlands; 4 Radboudumc Center for Mindfulness, Department of Psychiatry, Radboud UMC Nijmegen, 6500 HB Nijmegen, Netherlands

**Keywords:** Mindfulness, Experience sampling method, Nature, Exercise, Mood

## Abstract

The aim of this study was to explore the feasibility and effectiveness of mindful walking in nature as a possible means to maintain mindfulness skills after a mindfulness-based cognitive therapy (MBCT) or mindfulness-based stress reduction (MBSR) course. Mindful walking alongside the river Rhine took place for 1, 3, 6, or 10 days, with a control period of a similar number of days, 1 week before the mindful walking period. In 29 mindfulness participants, experience sampling method (ESM) was performed during the control and mindful walking period. Smartphones offered items on positive and negative affect and state mindfulness at random times during the day. Furthermore, self-report questionnaires were administered before and after the control and mindful walking period, assessing depression, anxiety, stress, brooding, and mindfulness skills. ESM data showed that walking resulted in a significant improvement of both mindfulness and positive affect, and that state mindfulness and positive affect prospectively enhanced each other in an upward spiral. The opposite pattern was observed with state mindfulness and negative affect, where increased state mindfulness predicted less negative affect. Exploratory questionnaire data indicated corresponding results, though non-significant due to the small sample size. This is the first time that ESM was used to assess interactions between state mindfulness and momentary affect during a mindfulness intervention of several consecutive days, showing an upward spiral effect. Mindful walking in nature may be an effective way to maintain mindfulness practice and further improve psychological functioning.

## Introduction

The psychological effects of the 8-week mindfulness-based stress reduction (MBSR) and mindfulness-based cognitive therapy (MBCT) are getting increased attention in scientific research, with mindfulness techniques being recommended as valuable strategies to relate to negative thoughts and emotions. Mindfulness training aims to make participants more aware of their automatic reactions on a behavioral, emotional, and cognitive level, by encouraging them to engage in daily meditation practices. Non-judgmental observing of emotions or thoughts present here and now enables participant to react in a calm and “wise” manner in stressful situations (Kabat-Zinn 1994). Mindfulness has been shown to lead to improvements in depression, anxiety, stress, and quality of life in a variety of clinical populations as well as healthy participants (Gotink et al. 2015).

Although studies directly examining the necessity of ongoing practice are lacking, mindfulness participants are recommended to maintain mindfulness practice in order to maximize and sustain benefits. As many find it difficult to do this on their own, mindfulness centers often offer reunion meetings or silent days. Attendees have reported to experience these as a booster reminding them of their mindfulness practices and as a sanctuary where these practices are further nurtured (Hopkins and Kuyken 2012). So, it might be valuable to offer other easily accessible means to former participants of MBCT or MBSR courses to help maintaining their mindfulness practice.

One possibility that is available to many people is mindful walking. During the MBCT and MBSR training, participants are asked to pay attention to their senses (i.e., sight, hearing, smelling, tasting, bodily sensations), emotions, thoughts, and automatic behavioral patterns. They are encouraged to experiment with letting go of automatic behaviors (i.e., high demands, hurrying) and with approaching difficult emotions or situations (i.e., annoyance, sadness). During the training, participants get introduced to mindful walking, which has been described as “meditation in motion.” Mindful walking uses the everyday activity of walking as a mindfulness practice to become more aware of bodily sensations. The physical sensation of walking enables people to feel more “grounded” in the present moment (Segal, Williams, and Teasdale 2002). When people’s minds are occupied or stressed, paying attention to their physical movements can be an easy way to become more mindful.

Walking does not require expensive gear or tools, and it can usually be integrated into a person’s schedule without too many difficulties. Mindfulness and walking share similar experiences. Both require paying attention to bodily sensations and other sensory input like sights, sounds, or smells. In addition to the intrinsic link between mindfulness and walking, physical exercise in itself has a positive influence on psychological functioning and can reduce depressive symptoms (Nabkasorn et al. 2006; Salmon 2001; Scully, Kremer, Meade, Graham, and Dudgeon 1998). A recent meta-analysis in the UK shows that outdoor walking can lead to lower blood pressure, resting heart rate, body fat, body mass index, total cholesterol and depression, and increased VO2max and physical functioning (Hanson and Jones 2015). Other studies on environment and mental health have shown the benefits of walking in nature (Easley, Passineau, and Driver 1990; Hartig, Mang, and Evans 1991; Kaplan and Talbot 1983; Roe and Aspinall 2011). In summary, mindful walking in nature could contribute to keeping up both mindfulness practice as well as a healthy lifestyle, thereby reducing stress-related symptoms and diseases.

Little is known about how exactly mindful walking may affect mindfulness and psychological functioning. One method to measure relationships between mental states in a detailed way is experience sampling method (ESM). ESM enables repeated in-the-moment assessments of experiential states to measure detailed fluctuations in participants (Csikszentmihalyi and Larson 1987; Peeters, Nicolson, Berkhof, Delespaul, and deVries 2003), thereby minimizing retrospective bias and enhancing reliability and ecological validity (Csikszentmihalyi and Larson 1987). Earlier ESM studies found that mindfulness stimulates upward spirals of positive affect and positive cognition (Garland, Geschwind, Peeters, and Wichers 2015), and that induced mindfulness enhanced valence and calmness, where induced rumination reduced these states (Huffziger et al. 2013). Furthermore, Snippe, Nyklíček, Schroevers, and Bos (2015) investigated daily affect changes during an MBSR training, and reported that changes in mindfulness preceded changes in affect.

This pilot study aims to explore the feasibility and effectiveness of mindful walking as a way to maintain mindfulness skills in people who previously participated in either an MBCT or MBSR course. Using ESM data, we tested whether mindful walking has beneficial effects on momentary positive and negative affect, allowance of thoughts and emotions, and state mindfulness and compared to a control period. With time-lagged analyses, we also investigated temporal relations between state mindfulness (at time *t*−1) and momentary positive and negative affect (at time *t*), and vice versa. Finally, we tested whether walking more days leads to added benefits, compared to the first day of walking. On an exploratory base, we used questionnaires to see whether ESM results corresponded with changes in self-reported depression, anxiety, stress, brooding, and mindfulness skills. The current study is the first to investigate the relationship between state mindfulness and momentary affect with ESM during a multi-day mindful walking intervention.

## Method

### Participants

All adult participants who had previously completed either an MBSR or an MBCT course at the Radboudumc Centre for Mindfulness in Nijmegen, the Netherlands, were eligible to participate in the study. There were no restrictions in terms of age, current psychopathology, or duration since completion of the mindfulness course. Of the 49 people attending the organized information meeting, 32 chose to participate in the study. Three participants were excluded from analyses due to acquired brain damage associated with cognitive impairments, which raised uncertainty about whether they could properly complete the assessments. The remaining 29 participants were on average 54.3 years old (SD 9.0) and 69 % were female (see Table 1). Six persons had participated in an MBSR course (general population) and 23 in an MBCT course (mainly patients with recurrent depressive disorder). Participation in these courses took place 2.7 years (SD 1.5) before the study. Many of the participants regularly attended reunion meetings at the Radboudumc Centre for Mindfulness. Ten people (34.5 %) participated in the 1-day walking retreat, 14 (48.3 %) in the 3-day walking retreat, and five (17.2 %) walked for six or more days.

**Table 1:** Characteristics of study participants

Participants	1 day	3 days	6 or more days
(*N* = 29)	*N* = 10 (34 %)	*N* = 14 (48 %)	*N* = 5 (17 %)
Age (SD)	55.1 (7.5)	52.5 (9.1)	57.9 (12.5)
Female	9 (90 %)	10 (71 %)	1 (20 %)
Education			
Lower education	3 (30 %)	4 (29 %)	1 (20 %)
Higher education	7 (70 %)	10 (71 %)	4 (80 %)
Living situation			
Alone	2 (20 %)	6 (43 %)	2 (40 %)
Partner	2 (20 %)	3 (21 %)	2 (40 %)
Partner and children	6 (60 %)	5 (36 %)	1 (20 %)
History of depression	5 (50 %)	14 (100 %)	4 (80 %)

### Procedure

By means of the center’s website, a Facebook group, and on reunion days at the center, adult participants of previous training groups were invited to an informal meeting at which they were informed about both the mindful walking period itself and the associated self-report measures and experience sampling. After the meeting, people gave their definitive consent to participate. For participants of the 1 and 3-day walking periods, all costs were reimbursed. Participants of the six or more days walking periods received €50 per day as a compensation for their accommodation costs. Participants were provided with smartphones to complete the experience sampling for ten quasi-random times a day both during the mindful walking period and control period (five times a day for those walking six or more days). In addition, participants were asked to complete self-report questionnaires before and after both the mindful walking period, and the control period of similar duration exactly 1 week before. As participants were no (longer) patients of the Radboud University Medical Center and the intervention and assessments were not considered to represent any risk to the participants, the medical ethics committee of the hospital waived the need to apply for formal ethical permission (CMO registration no. 2014/250).

The walking route took place alongside the river Rhine, from where it enters the Netherlands to where it joins the North Sea, reaching a total length of 240 km. The route was mostly unpaved, and participants were instructed to keep the river on their right and to overcome obstacles they might come across, such as fences and detours, in their own way. They received a map with a rough description of the route and available accommodation addresses.

People were offered three different possibilities to participate in the mindful walking: either as a 1-day walking retreat in a group, accompanied by a mindfulness teacher; or a 3-day walking retreat in a group, accompanied by two mindfulness teachers; or a solitary walking retreat of 6 days or more, with the possibility of daily contact with a mindfulness teacher by telephone, text, or email. Participants were instructed to walk the route silently except for times conversation was necessary for practical purposes (i.e., arrival at accommodation). They were reminded to pay attention to their senses (i.e., sight, hearing, smelling, tasting, bodily sensations), emotions, thoughts, and automatic behavioral patterns. They were encouraged to experiment with letting go of automatic behaviors (i.e., high demands, hurrying) and with approaching difficult emotions or situations (i.e., annoyance, sadness). They were also encouraged to choose wisely between walking alongside the river, the grass bank or the surfaced road on the dyke. For the 1-day walking retreat, there was a 15-min sitting meditation at the start of the day, at lunchtime, and at the end of the day. For the 3-day walking retreat, participants were offered standing yoga exercises for 30 min before breakfast, a sitting meditation of 30 min followed by a plenary enquiry in the evening and the possibility of individual interviews with one of the mindfulness teachers. The participants walking for six or more days walked on their own and hence only received the general walking meditation instructions. They were, however, encouraged to spend at least half an hour every day to write about their experiences and process in a personal diary. They also had the possibility of individual interviews at the end of the day to discuss their process with a mindfulness teacher.

The control period was of similar duration as the mindful walking period the participant had opted for and took place exactly 1 week before the intervention period, i.e., when the mindful walking period was from Friday to Sunday, the control period was from Friday to Sunday as well. The participants received no particular instructions about what to do during the control period.

### Measures

The assessments consisted of both electronic brief questions using the ESM at quasi-random times during the control and intervention periods, and self-report questionnaires before and after the control and mindful walking periods.

To closely monitor the course of participants’ experiential state, we collected ESM data using smartphones that quasi-randomly gave a signal several times a day between 9 a.m. and 4 p.m. to cover the period of mindful walking, with a maximum time in between of 90 min. For the 1- and 3-day walking periods, samples were taken ten times a day, and for the six and more day retreats, five times a day. After each signal, participants answered 18 Likert scale items each ranging from 0 (not at all) to 7 (very much). All items are listed in Table 2. The first nine items comprised different moods (Watson, Clark, and Tellegen 1988) with five items measuring positive affect (content, cheerful, relaxed, energetic, and calm) and four negative affect (sad, irritated, insecure, tense). The selection of affect items was based on previous experience sampling studies in this area of research (Geschwind, Peeters, Drukker, van Os, and Wichers 2011; Wichers et al. 2010). Cronbach’s alpha of the positive affect items was 0.92 and of negative affect items 0.89. The items on affect were followed by four items measuring suppressing or allowing negative thoughts and emotions. These items were combined into one outcome “allowing,” indicating the ability to allow negative thoughts and emotions, and not suppress them. Cronbach’s alpha of these four items was 0.79. Finally, participants answered the items with the highest factor loadings on four of the five subscales of the Five Facet Mindfulness Questionnaire (FFMQ) (Baer et al. 2008), namely Observing, Acting with awareness, Non-judgment, and Non-reacting. An item on self-compassion was added instead of an item for Describing, which has not been found influential in previous research (Schroevers and Brandsma 2010). The Cronbach’s alpha of these five items was 0.80.

**Table 2:** ESM items and Cronbach’s alpha

Outcome	Item	Cronbach’s alpha
Positive affect	Right now, how cheerful are you?	0.92
	Right now, how content are you?	
	Right now, how energetic are you?	
	Right now, how calm are you?	
	Right now, how relaxed are you?	
Negative affect	Right now, how sad are you?	0.89
	Right now, how insecure are you?	
	Right now, how irritated are you?	
	Right now, how tense are you?	
Allowing	My thoughts will not leave me alone	0.79
	I try to ignore my thoughts	
	My feelings carry me away	
	I try to suppress my feelings	
Mindfulness	I pay attention to sensations, such as the wind in my hair or sun on my face	0.80
	I perceive my feelings and emotions without having to react to them.	
	When I have distressing thoughts or images, I just notice them and let them go.	
	I find it difficult to stay focused on what’s happening in the present.	
	I am friendly and kind to myself	

All Likert scale items ranged from 1 (not at all) to 7 (very much). Cronbach’s alphas are based on between-subject analyses

All items not filled in within 15 min after the signal were excluded from analysis, as reports completed after this interval are considered less reliable and less ecologically valid (Delespaul 1995). Experience sampling data (control or intervention period) with fewer than 33 % valid reports were also excluded from the analysis.

The Dutch version of the Depression Anxiety Stress (DASS-21) questionnaire was used to measure depression, anxiety, and stress. The three subscales each consist of seven items scored on a Likert scale ranging from 0 (never) to 3 (very often). The DASS-21 has good validity and reliability in a non-clinical sample (Henry and Crawford 2005); Cronbach’s alpha in this sample was 0.90. The brooding subscale of the 22-item Ruminative Response Scale (RRS) (Schoofs, Hermans, and Raes 2010) was used to assess rumination. This scale consists of five items scored on a Likert scale ranging from 0 (never) to 3 (very often). Cronbach’s alpha of this subscale was 0.84.

As the pre and post assessments were so close together, particularly during the 1-day walking retreat, we selected a measure assessing mindfulness as a state rather than a trait, i.e., the subscales Decentering and Curiosity of the Toronto Mindfulness Scale (Lau et al. 2006). These two subscales are significantly and positively correlated with absorption and awareness of one’s surroundings. Curiosity is also significantly correlated with awareness of internal states (thoughts and feelings). The 13 items are scored on a five-point Likert scale ranging from 0 (not at all) to 4 (very much so). Their Cronbach’s alpha was 0.81.

### Data analyses

All analyses were performed in SPSS 22.0 (IBM Corp 2013). The approach used for the ESM data was the following: ESM data have a hierarchical structure. Thus, multiple observations (level 1) are clustered within participants (level 2). Mixed effect multilevel analyses with random (i.e., participant-specific) intercepts were used in order to take the variability associated with each level of nesting into account (Snijders 2012). On top of the random intercept, all time-lagged analyses used autocorrelated covariance structures with a random slope.

In the questionnaire data, 6.9 % of values were missing, and these were imputed based on multiple imputation regression with ten iterations. ANCOVA analyses describe the levels of dependent variables depression, anxiety, stress, brooding, mindfulness decentering, and mindfulness curiosity at each time point adjusted for gender, clinical indication, and number of days walked. This way the effect of the intervention could be observed more accurately, as gender, clinical indication and number of days walked may influence the outcome. In order to compare the different outcome measures, Cohen’s *d* for within-subject measurements (Morris and DeShon 2002) was calculated by comparing the mean delta of the pre-measurement with the mean delta of the post-measurements (based on the ANCOVA results). In other words, Cohen’s *d* indicates whether the change during the walking period was larger than during the control period (see Table 4).

## Results

In addition to the three cognitively impaired participants, data from two more participants were excluded from the ESM analysis because the proportion of valid assessments was less than 33 %. The baseline assessment of one participant was excluded from the analyses due to procedural problems during data collection; the data of the intervention period were still used.

The results of the regression analyses are presented in Table 3. Compared to the control period, the mindful walking periods resulted in a significant increase of positive affect (*β* = 0.91, *p* < 0.001) and state mindfulness (*β* = 0.98, *p* < 0.001), and a reduction of negative affect (*β* = −0.71, *p* < 0.001). Allowing thoughts and emotions showed only a trend of effect (*β* = 0.43, *p* = 0.067). Positive affect and mindfulness were highly correlated (*r* = 0.75, *p* < 0.001), and there was a negative correlation between negative affect and mindfulness (*r* = −0.66, *p* < 0.001). State mindfulness in the previous moment appeared to predict positive affect in the next, even after controlling for affect at the previous moment (*β* = 0.18, *p* < 0.001). Similarly, positive affect at the previous moment appeared to predict mindfulness in the next, even after controlling for mindfulness at the previous moment (*β* = 0.21, *p* < 0.001). Negative affect showed an opposite relationship: after controlling for the previous level, state mindfulness predicted a reduction of negative affect in the next moment (*β* = −0.14, *p* < 0.001) and negative affect predicted a reduction of mindfulness in the next moment (*β* = −0.19, *p* < 0.001).

**Table 3:** Effect estimates of hypotheses tested in ESM data analysis

							95 % CI		
Hypothesis	Outcome	Parameter		*β*	SE	*p* value	Lower		Upper
Effect of the intervention	Positive affect	Intercept		4.27	0.15	<0.001	3.97		4.57
		Intervention		0.91	0.21	<0.001	0.48		1.33
	Negative affect	Intercept		2.56	0.13	<0.001	2.29		2.82
		Intervention		−0.71	0.19	<0.001	−1.08		−0.34
	Mindfulness	Intercept		4.24	0.15	<0.001	3.94		4.54
		Intervention		0.98	0.21	<0.001	0.56		1.40
	Allowing	Intercept		5.06	0.16	<0.001	4.73		5.39
		Intervention		0.43	0.23	0.067	−0.03		0.90
Time-lagged analyses: effect of previous moment on following moment^c^	Positive affect	Intercept		1.39	0.12	<0.001	1.15		1.63
		Previous mindfulness		0.18	0.03	<0.001	0.12		0.25
		Previous positive affect		0.53	0.03	<0.001	0.47		0.60
		Interaction terms^a^				NS			
	Negative affect	Intercept		1.62	0.23	<0.001	1.17		2.07
		Intervention		0.10	0.31	NS	−0.52		0.71
		Previous mindfulness		−0.14	0.04	<0.001	−0.21		−0.07
		Previous negative affect		0.59	0.04	<0.001	0.52		0.67
		Interaction terms^b,d^		−0.16	0.05	0.002	−0.27		−0.06
	Mindfulness	Intercept		1.75	0.13	<0.001	1.49		2.02
		Previous mindfulness		0.43	0.03	<0.001	0.36		0.49
		Previous positive affect		0.21	0.03	<0.001	0.14		0.27
		Interaction terms^a^				NS			
	Mindfulness	Intercept		2.91	0.21	<0.001	2.50		3.31
		Previous mindfulness		0.48	0.03	<0.001	0.42		0.54
		Previous negative affect		−0.19	0.03	<0.001	−0.25		−0.12
		Interaction terms^b^				NS			
Time-lagged analyses: effect of previous on following day	Positive affect	Intercept		2.18	0.64	0.001	0.90		3.47
		Mindfulness previous day		0.23	0.17	0.192	−0.12		0.58
		Positive affect previous day		0.36	0.19	0.060	−0.02		0.74
	Negative affect	Intercept		1.74	0.91	0.063	−0.10		3.58
		Mindfulness previous day		−0.11	0.13	0.411	−0.38		0.16
		Negative affect previous day		0.38	0.15	0.012	0.09		0.68
	Mindfulness	Intercept		3.50	0.53	0.005	1.91		5.09
		Mindfulness previous day		0.66	0.17	0.002	0.29		1.03
		Positive affect previous day		−0.36	0.12	0.027	−0.65		−0.06
	Mindfulness	Intercept		0.86	1.10	0.435	−1.30		3.02
		Mindfulness previous day		0.67	0.15	<0.001	0.31		1.03
		Negative affect previous day		0.41	0.13	0.002	0.15		0.67
Effect of extra days walking	Positive affect	Intercept		4.89	0.19	<0.001	4.51		5.27
		Day		0.12	0.04	0.011	0.03		0.20
	Negative affect	Intercept		2.04	0.16	<0.001	1.72		2.37
		Day		−0.04	0.04	0.286	−0.12		0.04
	Mindfulness	Intercept		4.92	0.16	<0.001	4.58		5.27
		Day		0.14	0.00	<0.001	0.14		0.14

*95 % CI* 95 % confidence interval, *SE* standard error, *NS* not statistically significant

^a^Interaction terms positive affect: previous mindfulness × intervention, previous positive affect × intervention

^b^Interaction terms negative affect: previous mindfulness × intervention, previous negative affect × intervention

^c^Interaction with group condition was non-significant; therefore, data were collapsed across the control and intervention period

^d^Interaction term intervention × previous negative affect was significant; therefore, split file on condition was performed

**Table:** 

Split file	Parameter	*β*	SE	*p* value	Lower	Upper
					95 % CI	95 % CI
Intervention group	Intercept	1.99	0.26	<0.001	1.49	2.50
	Negative affect previous moment	0.41	0.04	<0.001	0.32	0.49
	Mindfulness previous moment	−0.17	0.04	<0.001	−0.25	−0.10
Control group	Intercept	1.88	0.25	<0.001	1.39	2.38
	Negative affect previous moment	0.48	0.04	<0.001	0.39	0.56
	Mindfulness previous moment	−0.14	0.04	0.001	−0.22	−0.06

Outcome: Negative affect

Mindfulness on the previous day did not predict positive affect on the next day, even after controlling for positive affect on the previous day (*β* = 0.23, *p* = 0.192), nor did mindfulness on the previous day predict a reduction of negative affect on the next day (*β* = −0.11, *p* = 0.411). In contrast with the momentary analyses, positive affect on the previous day did significantly predict a reduction of mindfulness on the next day when controlling for the level of mindfulness on the day before (*β* = −0.36, *p* = 0.027), and negative affect on the previous day predicted an increase of mindfulness the next day (*β* = 0.41, *p* = 0.002). Both state mindfulness and positive affect appeared to significantly improve with the number of days walked (*β* = 0.14, *p* < 0.001, and *β* = 0.12, *p* = 0.011, respectively), but negative affect did not (*β* = −0.04, *p* = 0.286).

Because the questionnaire data are underpowered, we present Cohen’s *d* to give an indication of the effect size, rather than focusing on statistical significance. During the control period (between measurements 1 and 2), there were no consistent or large changes from pre to post (Table 4). During the intervention period (between measurements 3 and 4), depression, anxiety, stress, brooding, and mindfulness all improved moderately (Cohen’s *d* ranged from 0.27 for depression to 0.53 for brooding), but compared to the control period these changes were not statistically significant.

**Table 4:** Questionnaire data: ANCOVA repeated measures analysis, delta within control period and intervention period, and standardized effect size (Cohen’s *d*)

	Time	Mean (95 %CI)	Δ	Cohen’s *d* (95 % CI)
Depression	1	6.7 (5.3–8.2)	−0.6	0.27 (−0.26 to 0.79)
	2	6.1 (4.6–7.5)		
	3	5.7 (4.2–7.1)	−1.8	
	4	3.9 (2.5–5.4)		
Anxiety	1	5.1 (4.2–6.1)	0.0	0.45 (−0.08 to 0.98)
	2	5.1 (4.1–6.0)		
	3	4.6 (3.6–5.5)	−1.7	
	4	2.9 (2.0–3.8)		
Stress	1	9.9 (8.6–11.2)	−0.5	0.49 (−0.05 to 1.02)
	2	9.4 (8.1–10.7)		
	3	9.3 (8.0–10.6)	−3.0	
	4	6.3 (5.0–7.6)		
Brooding	1	6.4 (5.3–7.5)	+0.3	0.53 (−0.006 to 1.06)
	2	6.7 (5.6–7.8)		
	3	6.3 (5.3–7.4)	−1.7	
	4	4.6 (3.4–5.6)		
Mindfulness Decentering	1	12.6 (11.2–14.0)	−0.5	0.53 (−0.005 to 1.06)
	2	12.1 (10.7–13.6)		
	3	13.2 (11.8–14.6)	+2.2	
	4	15.4 (14.0–16.8)		
Mindfulness Curiosity	1	14.8 (13.2–16.4)	−0.1	0.20 (−0.32 to 0.73)
	2	14.7 (13.1–16.4)		
	3	16.0 (14.3–17.6)	+0.9	
	4	16.9 (15.2–18.5)		

*N* = 29. Time 1 (pre) and 2 (post) represent the control period. The intervention took place between time 3 (pre) and time 4 (post). Analyses are adjusted for sex, number of days walked, and MBSR/MBCT. A positive Cohen’s *d* indicates a stronger improvement on the outcome measure during the intervention than during the control period

*95 % CI* 95 % confidence interval

## Discussion

This pilot study explored the feasibility and effectiveness of mindful walking as a way of maintaining practice after having participating in an MBCT or MBSR course, using standard questionnaires as well as experience sampling. The ESM data shows that mindful walking resulted in significant improvements of both mood and mindfulness skills. Moreover, the ESM data provided more detailed insight in how the moment-to-moment changes in mood and mindfulness affect each other during the mindful walking period: an earlier randomized controlled trial showed that MBCT increases positive affect as well as individual’s susceptibility to rewarding situations in daily life (Geschwind, Peeters, Huibers, van Os, and Wichers 2012). The current data corroborate and extend this finding by showing that state mindfulness and positive affect, measured during a mindfulness intervention which covered a period of consecutive days, appear to stimulate each other. Increased state mindfulness predicted a more positive affect in the next moment and less negative affect, which in turn predicted a higher state mindfulness, thereby creating an upward spiral from one moment to the next. The moment-to-moment ESM data have enough power to monitor this relationship. The less well-powered day-to-day data show a different trend, in the sense that mindfulness did not predict either positive or negative affect, and positive affect seemed to predict less mindfulness and negative affect more. It could be that over such a period, having a good day slackens mindfulness practice on the next, where having more negative feelings increases practice the next day, but these findings need more replication in a larger sample before any conclusions can be drawn.

Our findings are in accordance with previous research showing that changes in mindfulness precede changes in affect (Snippe et al. 2015). Garland and colleagues compared interactions between positive affect and cognitions before and after MBCT and found that MBCT appeared to enhance upward spirals between positive affect and cognition (Garland et al. 2015). While Garland and Snippe investigated the influence of mindfulness training on mood, we investigated the moment-to-moment temporal relationships between mindfulness and mood. Our method of analysis adheres to state of the art principles in multilevel time-series modeling, which has also been used in previous high-impact publications (Collip et al. 2013; Geschwind et al. 2011; Snippe et al. 2015). Regarding the (underpowered) self-report questionnaires, the results were in line with the ESM outcomes: mindful walking can reduce depression, anxiety, stress, and brooding and increase mindfulness skills, although these changes did not reach statistical significance compared to a control period of similar length.

Although the current study offers promising results, there are several limitations that have to be taken into account. First, this is a small sample of self-referred participants. Chances are that there is a selection bias of people who benefitted from the mindfulness training or believe in the effect of walking in nature, and thus were more motivated to participate in this study. As the study population was limited to people who previously participated in MBCT of MBSR, it is not sure what the effectiveness of the mindful walking would have been in people who do not have that experience. Our choice of restricting the study population to graduates of the mindfulness courses was influenced by the fact that we anticipated that being in silence for prolonged periods of time would not be easy for people who are naive to meditation. We also anticipated that it would not have been easy for them to pay non-judgmental attention to bodily sensations, emotions, and thoughts, to become aware of automatic patterns and start experimenting with alternative ways of dealing with situations without any further guidance. However, as the mean time since completion of the MBCT or MBSR course was several years for most participants, it remains unsure whether the effectiveness of the mindful walking was due to consolidating prior practice or to being offered something new. Many of the participants, though, had been maintaining their mindfulness skills by attending the reunion meetings of the Radboudumc Centre for Mindfulness.

With regard to the small sample size, the questionnaire results have been more affected than the ESM data due to the number of repeated measurements per person. In addition, although pragmatic, using subscales of validated questionnaires may reduce validity and reliability of our questionnaire measurements (Breakwell, Smith, and Wright 2012). Also, while most participants previously participated in an MBCT group for their recurrent depression, no formal psychiatric assessment has been done before inclusion of participants, so apart from demographic information unfortunately no further information about current psychopathology is available.

Although the study included a control period, it did not include a control group. Consequently, it remains unclear if similar effects would be evident with any other activity in a group or in nature. Furthermore, the intervention varied in length and in presence of a group leader. As the intervention consisted of a combination of mindfulness practice and physical exercise, it also remains unclear to what extent either of these is helpful. Possibly, monitoring of physical exercise in addition to mood and mindfulness skills in a next study might enable us to further disentangle this process. Finally, the study did not include a follow-up period, preventing conclusions about the consolidation of the treatment effects.

The results of this study should be regarded as preliminary. Replication in a properly powered randomized controlled trial, including a control group and a follow-up period, is necessary. The ESM data, however, give us a glimpse of a possible working mechanism between mindfulness and enhanced well-being, through upward spiral effects between state mindfulness and improved mood from one moment to the next.
